# A silent gap: underrepresentation of Latin American patients in pivotal genitourinary and skin cancer trials

**DOI:** 10.1093/oncolo/oyag259

**Published:** 2026-08-03

**Authors:** Erika Adriana Martinez-Castaneda, Ava Isabella De Pellegrin Overgaard, Jean Henri Maselli-Schoueri, Jennifer A H Bell, Srikala S Sridhar

**Affiliations:** Division of Medical Oncology and Hematology, Princess Margaret Cancer Centre, University of Toronto, Toronto, ON M5G 2C4, Canada; Division of Medical Oncology and Hematology, Princess Margaret Cancer Centre, University of Toronto, Toronto, ON M5G 2C4, Canada; Division of Medical Oncology and Hematology, Princess Margaret Cancer Centre, University of Toronto, Toronto, ON M5G 2C4, Canada; Princess Margaret, Department of Supportive Care Research DivisionToronto, M5G 2C4, Canada; UHN Department of Clinical and Organizational Ethics; University of Toronto, Department of PsychiatryToronto, M5G 2C4, Canada; Division of Medical Oncology and Hematology, Princess Margaret Cancer Centre, University of Toronto, Toronto, ON M5G 2C4, Canada

**Keywords:** global oncology, Latin America, genitourinary cancer, melanoma and skin cancer, health disparities, ethics

## Abstract

**Background:**

Latin America bears a growing burden of genitourinary (GU) and cutaneous malignancies but remains underrepresented in clinical trials that shape global standards of care. This underrepresentation raises ethical concerns and limits the generalizability of trial findings. We evaluated Latin American (LATAM) representation in pivotal GU and skin cancer trials and explored barriers to equitable inclusion.

**Methods:**

We reviewed pivotal phase II and III trials in advanced GU and cutaneous malignancies (melanoma and non-melanoma skin cancers) published between 2015 and 2024. Extracted data included trial site distribution, geographic representation, and ethnicity reporting.

**Results:**

A total of 46 pivotal trials (29 GU and 17 skin cancer trials), representing 38 900 patients, were included. LATAM trial sites were present in 44 studies; however, only 25 trials reported enrollment of Hispanic/Latino patients, totaling 1303 patients (3.35%). Representation across LATAM was highly concentrated in a small number of countries, including Brazil (16 trials), Argentina (14), Chile (13), Mexico (12), and Colombia (6). Participation was greater in GU trials compared with skin cancer trials. Reporting transparency was limited: Hispanic/Latino representation was reported in only 2 main publications, 5 supplementary appendices, and 23 ClinicalTrials.gov records.

**Conclusions:**

Only 6.3% of participants in pivotal GU and skin cancer trials were from Latin America. Limited infrastructure, regulatory barriers, and restricted research investment likely contribute to this disparity. Improving equitable representation will require intentional trial design, stronger regional investment, and policies that move beyond nominal participation toward meaningful inclusion.

## Introduction

By 2030, nearly 75% of all cancer-related deaths are projected to occur in low- and middle-income countries (LMICs). However, cancer research remains disproportionately concentrated in high-income countries (HICs), limiting the generalizability and global applicability of clinical trial evidence.^1^^,^^2^ Latin America (LATAM), composed predominantly of upper-middle–income countries, exemplifies this broader LMIC related gap, with a growing cancer burden but persistent underrepresentation in the clinical trials that inform global standards of care.

Given that global oncology trials must include a meaningful proportion of participants from LMIC regions to adequately assess both treatment efficacy and applicability, and that data remain particularly scarce for malignancies with rapidly increasing incidence in these settings, including genitourinary (GU) and skin cancers, the aim of this study was to assess the actual inclusion of Latin American participants in recent practice changing clinical trials.^3^^,^^4^

## Methods

We reviewed practice changing phase II/III trials in advanced GU and cutaneous skin cancers (melanoma, non-melanoma) published between 2015 and 2024. Data extracted included geographic trial participation, country site distribution, and reporting of Hispanic/Latino ethnicity. Descriptive analyses were used to characterize the absolute numbers and rates of participation of Latin American participants.

## Results

A total of 46 pivotal trials (29 GU, 17 Skin) enrolling 38 900 patients were analyzed. Latin America sites appeared in 44 of 46 trials, however only 25 trials enrolled Hispanic/Latino patients (*n* = 1303 patients; 3.35%). All GU trials referenced Latin America participation, but enrollment was inconsistent. Among melanoma trials, 15 of 17 (88%) mentioned Latin America involvement, while 2 did not.

Geographic participation was highly concentrated, with enrollment limited to five countries: Brazil (16 trials), Argentina (14), Chile (13), Mexico (12), and Colombia (6). Representation was higher in GU trials than in skin cancer studies. Transparency in ethnicity reporting was limited: only 2 trials reported Hispanic/Latino data in primary tables, 5 in supplementary materials, and 23 only in ClinicalTrials.gov. (Table 1).

**Table 1.** Latin American representation in pivotal genitourinary and skin cancer trials (2015-2024).

Characteristic	Number (%)
**Total trials analyzed**	46
**Total patients enrolled**	38, 900
**Trials with Latin American sites**	44 (96%)
**Trials enrolling Hispanic/Latino patients**	25 (54%)
**Hispanic/Latino patients enrolled**	1303 (3.35%)
**Trials reporting Hispanic/Latino ethnicity in main tables**	2 (4%)
**Trials reporting ethnicity only in supplements or ClinicalTrials.gov**	28 (61%)

## Discussion

These findings demonstrate that meaningful patient inclusion from Latin America remains minimal. When populations accounting for a substantial proportion of the global cancer burden are inadequately represented in key clinical trials, the resulting evidence base risks perpetuating disparities in access, outcomes, and treatment relevance. In Latin America, this underrepresentation limits the applicability of evidence used to define global standards of care and should not be viewed as unique to the region, but rather as part of a broader challenge affecting other historically underrepresented populations in global oncology research.

Limited access to trained investigators, research infrastructure, and regulatory support likely contributes to the exclusion of large segments of the Latin American population from global trials.

Site selection in multinational oncology trials is frequently driven by operational considerations, including anticipated recruitment rates, regulatory timelines, and prior research experience. Consequently, sponsors may preferentially select a limited number of established centers, potentially restricting broader regional participation despite substantial disease burden.

These observations are consistent with recent analyses of phase III oncology trials, which found that only 10 of 33 Latin American countries participated in global oncology studies and that more than 75% of regional trial sites were concentrated in Brazil, Argentina, and Mexico. Such geographic concentration suggests that disparities in research infrastructure, funding, investigator networks, and regulatory capacity continue to influence where clinical research is conducted across the region.^5^

Addressing linguistic barriers, health literacy, and culturally appropriate consent processes is also critical to improving participation. Achieving equitable representation will require coordinated institutional and policy level efforts to ensure that advances in cancer care benefit all populations.

Beyond these structural barriers, this gap highlights the relevance of bioethics as an analytical and methodological framework in global oncology research. Rather than reflecting a lack of ethical commitment among clinicians, bioethics offers tools to examine how trials are designed and translated across diverse populations. Integrating bioethics frameworks into oncology research training may support more inclusive and context sensitive trial designs.^6^^,^^7^

## Conclusions

The underrepresentation of LATAM participants in pivotal clinical trials reflects persistent structural barriers while also pointing to a broader ethical gap in global cancer research. Addressing this gap is essential to ensure that future oncology trials generate evidence that is both scientifically valid and globally applicable.

## Data Availability

The data underlying this article were derived from publicly available clinical trial publications and trial registries (including ClinicalTrials.gov). All data supporting the findings of this study are included within the article and its supplementary materials.
